# Global research landscape and emerging trends of non-coding RNAs in prostate cancer: a bibliometric analysis

**DOI:** 10.3389/fphar.2024.1483186

**Published:** 2025-01-07

**Authors:** Yu-Liang Zhou, Wen-Liang Yao, Sheng-Hui Chen, Peng Wang, Jing-Wen Fu, Jia-Qin Zhao, Jia-Yi Zhang

**Affiliations:** ^1^ Clinical School of Medicine, Jiangxi University of Chinese Medicine, Nanchang, Jiangxi, China; ^2^ Department of Andrology, Affiliated Reproductive Hospital of Jiangxi University of Chinese Medicine, Nanchang, Jiangxi, China

**Keywords:** non-coding RNA, prostate cancer, bibliometric analysis, extracellular vesicle, circular RNA

## Abstract

**Background:**

Prostate cancer (PC) is the most frequently diagnosed cancer in men and continues to be a major cause of cancer-related mortality worldwide. In recent years, non-coding RNAs (ncRNAs) have emerged as a significant focus in molecular biology research, playing a pivotal role in the development and progression of PC. This study employed bibliometric analysis to explore the global outputs, research hotspots, and future trends in ncRNA-related PC research over the past 20 years.

**Methods:**

Publications on PC-related ncRNAs from 2004 to 2023 were retrieved from Web of Science Core Collection. The co-operation network of countries, institutions, and authors on this topic was analyzed using CiteSpace (version 6.2. R6). In addition, co-occurrence analysis of keywords and co-citation analysis of references were performed using CiteSpace, and emergent detection was also performed.

**Results:**

A total of 2,951 articles on PC-related ncRNAs were finally included in this study for analysis. China contributed the largest number of publications, while the United States was the most influential country in this field, with collaborative ties to 26 other countries. Fudan University was identified as the most active institution in this field. Rajvir Dahiya was the most prolific and influential author. Within the co-citation network, clusters labeled “EVs,” “circRNA,” and “ceRNA” represented current research directions. The cluster labeled “gene” dominated the co-occurrence keywords. “circRNA” showed the highest burst strength among keywords, with “circRNA,” “EVs” and “exosome” maintaining sustained burst strength, suggesting these are the emerging research frontiers in this field.

**Conclusion:**

Investigating ncRNAs as pivotal research subjects in PC is essential for addressing the public health impact of the disease and advancing innovative diagnostic and targeted therapeutic strategies. This study provides a comprehensive bibliometric analysis of research related to PC-associated ncRNAs, delivering a scientific perspective and identifying potential research directions for scholars in this field.

## 1 Introduction

Prostate cancer (PC) is one of the most common malignancies threatening men’s health (Litwin and Tan, 2017). According to GLOBOCAN cancer statistics, PC had an estimated 1.5 million new cases and 397,000 deaths globally in 2022, making it the second most common cancer and the fifth leading cause of cancer-related mortality among men (Bray et al., 2024). As an age-related malignancy of the male genitourinary system, PC has a higher incidence in elderly populations and continues to rise with the increasing aging of the population (Siegel et al., 2023). Family history, race, and genetic syndromes are established risk factors for PC, while smoking and obesity have a suspected role in modulating PC-specific mortality (Gandaglia et al., 2021). In its early stages, PC is asymptomatic, with early detection primarily achieved through blood tests for prostate-specific antigen (PSA) and confirmed via tissue biopsy (Van Poppel et al., 2022). Although radiation therapy and radical prostatectomy are effective treatments for localized PC, they may increase the risk of treatment-related complications, including incontinence and erectile dysfunction from surgery, and gastrointestinal and erectile dysfunction from radiation therapy (Pinsky and Parnes, 2023). Androgen deprivation therapy (ADT) is the standard treatment for recurrent or metastatic PC patients, encompassing both surgical and pharmacological castration, with the latter being more commonly employed (Feldman and Feldman, 2001). The goal of ADT is to lower serum testosterone levels in PC patients and maintain this suppression to induce cancer cell death and achieve transient clinical remission (Schröder et al., 2012). Nevertheless, the vast majority of PC patients subjected to ADT will ultimately develop castration-resistant PC (CRPC), which is a leading cause of mortality among these patients (Cai et al., 2023).

For decades, the functional focus of PC research has primarily been on protein-coding genes. However, these genes constitute only about 2%–3% of the human genome, while the diverse and ubiquitous non-coding RNAs (ncRNAs) originate from the remaining nucleotides (Rinn and Guttman, 2014). With the advent of high-throughput sequencing technologies, most of the non-coding genome has been characterized as functional transcripts, playing crucial roles in various biological processes and pathological states (Slack and Chinnaiyan, 2019). ncRNAs are localized in both the nucleus and cytoplasm and can also be found within extracellular vesicles (EVs), such as exosomes, or other microvesicles in bodily fluids. Based on their length, ncRNAs can be broadly categorized into two main families: small ncRNAs, which are shorter than 200 nucleotides and include microRNAs, transfer RNA-derived small RNAs, and Piwi-interacting RNAs, and long ncRNAs (lncRNAs), which exceed 200 nucleotides and encompass pseudogenes, chimeric RNAs, and circular RNAs (circRNAs) (Brosnan and Voinnet, 2009; Shi et al., 2021). As competing endogenous RNAs (ceRNAs), ncRNAs interact with protein-coding messenger RNAs (mRNAs) in complex ways, offering new insights into the gene regulatory networks of humans. Notably, recent studies have demonstrated that certain ncRNAs, initially considered incapable of protein-coding, contribute critically to disease development and progression through the production of derived peptides or proteins (Wang et al., 2019; Yi et al., 2024). ncRNA therapies have progressed relatively slowly on a global scale, with most ncRNA-related drugs still in the early phases of human clinical trials. For example, a 13-mer locked nucleic acid (LNA) inhibitor of miR-221, known as LNA-i-miR-221, demonstrated promising anti-tumor activity and safety in a Phase I clinical study (Tassone et al., 2023). The miR-122 inhibitor miravirsen resulted in a dose-dependent reduction of hepatitis C virus RNA levels in a Phase II clinical trial (Janssen et al., 2013). Similarly, CDR132L, a miR-132 inhibitor, has demonstrated effectiveness in improving cardiac function in patients with heart failure (Täubel et al., 2021). Additionally, remlarsen (miR-29b mimic) was found to inhibit collagen expression and the development of fibroproliferation in incisional skin wounds among healthy volunteers (Gallant-Behm et al., 2019). Recent studies have extensively reported abnormal expression of ncRNAs as a significant feature of PC, identifying them as key players in the onset and progression of the disease (Ramalho-Carvalho et al., 2016; Mugoni et al., 2022). Research into the biology of ncRNAs not only reveals their potential roles in PC pathology but also provides a theoretical foundation for specific diagnostic, therapeutic, and preventive strategies targeting ncRNAs in the human genome.

Bibliometrics offers an objective approach for objectively reflecting the knowledge structure and emerging trends in a particular field through quantitative analysis of published scientific literature (Nicolaisen, 2010). CiteSpace, as a tool for visualizing scientific knowledge, aids researchers in examining contributions from authors, countries, and institutions, identifying rapidly evolving topics, and forecasting future research directions (Chen, 2004; Synnestvedt et al., 2005). For instance, Zhong et al. (2022) utilized bibliometric analysis to evaluate global scientific output on immunotherapy for PC from 1999 to 2021, summarizing future research trends. Similarly, Chen et al. (2022) investigated the research status, hotspots, and trends in bone metastases of PC through bibliometric analysis. Despite the rapid advancement of research on ncRNAs in recent years, the impact of these studies on the PC field has not yet been fully assessed. Therefore, this study aimed to employ bibliometric analysis and knowledge domain mapping to evaluate the publications on ncRNAs in PC published over the past 20 years in the Web of Science Core Collection (WoSCC), with the goal of identifying the knowledge domain and emerging trends in this field.

## 2 Methods

### 2.1 Data collection

**FIGURE 1:**
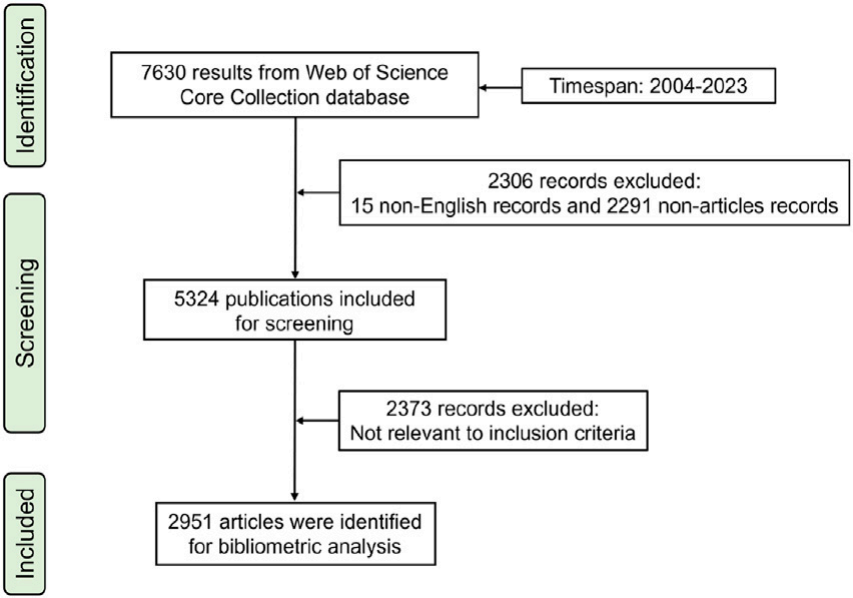
Flowchart of the selection process.

The Science Citation Index-Expanded (SCI-Expanded) of WoSCC was searched for publications related to ncRNAs in PC. The retrieval strategy used in this study was “TS = [(“miRNA*” OR “microRNA*” OR “miR-*” OR “lncRNA*” OR “long noncoding RNA*” OR “long non-coding RNA*” OR “circRNA*” OR “circular RNA*” OR “circ_*” OR “non-coding RNA*” OR “noncoding RNA*”) AND (“prostate cancer” OR “prostate neoplasm”)].” The search time span was set to between 1 January 2004 and 31 December 2023. In the first stage of screening, the type of publication was restricted to Article and the language was limited to English. In the second stage, irrelevant publications were excluded based on the title, keyword, abstract, and full text. The full records and cited references of eligible articles were exported from SCI-Expanded in plain text format. The flow chart of research steps of this study is shown in Figure 1.

### 2.2 Bibliometric analysis

All valid data were imported into Origin (version 2021) or CiteSpace (version 6.2. R6) for analysis and visualization. Origin was used to conduct statistical analysis on the annual number of publications and their citations, and to visualize the collaborations between different countries. CiteSpace was performed for co-operation analysis of countries, institutions, and authors. In addition, we used CiteSpace to draw a dual-map overlay of journals to investigate the evolutionary relationship between knowledge topics in directly citing journals and cited journals. In keyword analysis, we merged keywords with the same meaning to get a better perspective. The co-occurrence analysis of keywords and the co-citation analysis of references were performed by CiteSpace. Moreover, burst detection in CiteSpace was used to detect sudden surges in references and keywords at a certain time.

## 3 Results

### 3.1 Publication outputs and trends

**FIGURE 2:**
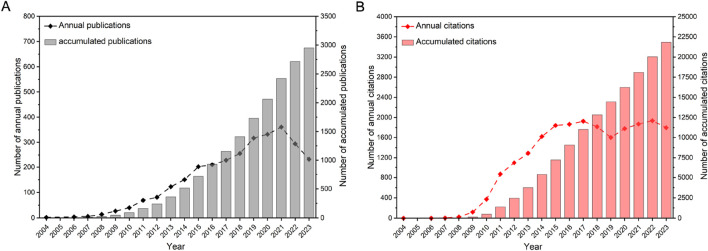
The distribution of publications **(A)** and their citations **(B)** from 2004 to 2023. The data were extracted using CiteSpace, and the figure was created with Origin.

A total of 2,951 articles on ncRNAs in PC were screened from 7,630 records retrieved from WoSCC. According to the number of citations, the total citations of the top 10 most cited papers accounted for 43.87% of all papers (Supplementary Table S1). The most cited article was titled “*c-Myc suppression of miR-23a/b enhances mitochondrial glutaminase expression and glutamine metabolism,*” which had 1,641 citations in *Nature*. The article published in *Cell* titled “*The Landscape of Circular RNA in Cancer*” had the highest annual citation frequency. The distribution of publications and their citations is presented in Figure 2. From 2004 to 2023, the cumulative number of global publications showed a consistent upward trend. Specifically, the evolution of PC-related ncRNA research consisted of threee stages. The field was in its early stages from 2004 to 2008, when fewer than 20 papers were published each year. The second stage was from 2009 to 2021, with a rapid increase in the number of annual publications. As the third stage from 2022 to 2023, the number of annual publications was in a steady growth, and the cumulative number of publications in 2023 was 113.5 times that of 2008. The cumulative number of citations for all articles in 2023 was 21,846, with an average of 7 citations per article. These results indicate that ncRNA research is an active field in PC and has received extensive attention from scholars.

### 3.2 Analysis of countries/regions and institutions

**FIGURE 3:**
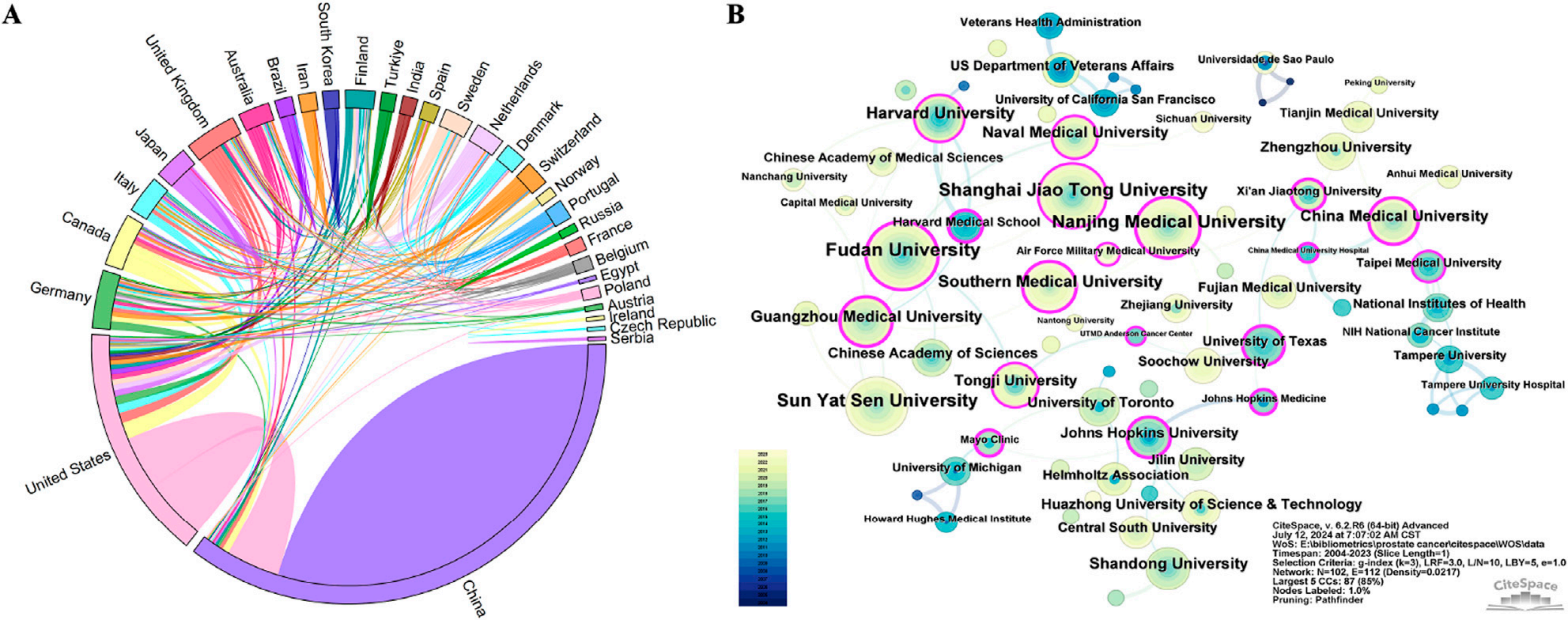
Collaboration network of countries/regions and institutions of ncRNA research in PC from 2004 to 2023. **(A)** The cooperation string diagram between top 30 countries/regions. **(B)** The network visualization map of different institutions. Each node represents an institution, and the size of the node corresponds to the frequency of publications produced by that institution. Each link between two nodes indicates a collaborative relationship between the respective institutions. Nodes with purple rings denote higher centrality, signifying their prominent role in the collaboration network. The data were extracted using CiteSpace. Panel **(A)** was created using Origin, while Panel **(B)** was generated with CiteSpace.

Analyzing the research output of countries/regions and institutions provides insights into the global distribution and trends within a specific field, enabling the identification of influential countries/regions and institutions. A total of 78 countries/regions were involved in the publications of ncRNAs in PC. Among them, China (1,665), the United States (699), Germany (131), Canada (120), and Italy (112) were the top five productive countries/regions in terms of the number of publications (Table 1). The H-index is a hybrid quantitative metric used to measure academic impact. The United States (119) has the highest H-index, indicating its leading academic influence in PC-related ncRNA research, followed by China (88), Canada (47), Japan (46), and Italy (43). Centrality helps identify key roles in information flow and knowledge dissemination within a network. The United (0.56) States exhibited the highest centrality, followed by China (0.27), the United Kingdom (0.2), Canada (0.11), and Germany (0.1). As depicted in Figure 3A, the United States collaborated with 26 other countries, notably including China, Canada, and the United Kingdom, which underscores its significant role as a central hub in international collaborations.

**TABLE 1:** Top 10 productive countries/regions of ncRNA research in PC from 2004 to 2023.

Rank	Country	Counts	Percentage	H-index	Centrality	Total citations
1	China	1,665	56.42	88	0.27	47,992
2	United States	699	23.69	119	0.56	53,405
3	Germany	131	4.44	42	0.1	6,767
4	Canada	120	4.07	47	0.11	7,106
5	Italy	112	3.80	43	0.07	6,487
6	Japan	100	3.39	46	0.02	8,339
7	United Kingdom	93	3.15	34	0.2	5,276
8	Australia	56	1.90	31	0.03	2,981
9	Brazil	52	1.76	25	0.05	1,920
10	Iran	52	1.76	16	0.09	882

The data were extracted using CiteSpace.

Based on the number of publications, Table 2 presents the top 10 institutions of ncRNA research in PC, with 9 of them located in China. Fudan University (98) had the highest number of publications, followed by Shanghai Jiao Tong University (96), Nanjing Medical University (91), and Sun Yat-sen University (91). Furthermore, the H-index of Sun Yat-sen University (36) and Harvard University (36) was the highest, followed by Fudan University (34) and Shanghai Jiao Tong University (33). Visualized with CiteSpace, we mapped an institutional collaboration network consisting of 102 nodes and 112 linkages (Figure 3B). Southern Medical University (0.41) had the highest centrality, followed by Guangzhou Medical University (0.36) and Nanjing Medical University (0.29). As the institution with the most publications, Fudan University primarily had close collaborations with Naval Medical University, Tongji University, and Chinese Academy of Sciences.

**TABLE 2:** Top 10 productive institutions of ncRNA research in PC from 2004 to 2023.

Rank	Institution	Country	Counts	H-index	Centrality	Total citations
1	Fudan University	China	98	34	0.24	3,472
2	Shanghai Jiao Tong University	China	96	33	0.14	3,297
3	Nanjing Medical University	China	91	30	0.29	3,096
4	Sun Yat-sen University	China	91	36	0.01	3,700
5	Southern Medical University	China	69	27	0.41	1,971
6	Harvard University	United States	63	36	0.15	4,674
7	Guangzhou Medical University	China	62	31	0.36	2,379
8	Tongji University	China	57	26	0.15	2,303
9	University of Toronto	Canada	55	30	0.09	2,834
10	Shandong University	China	55	24	0.03	1,640

The data were extracted using CiteSpace.

### 3.3 Analysis of authors

Author analysis facilitates the identification of pivotal contributors within a field and offers insights to guide academic exchanges and project collaborations. The top 10 active authors are listed in Table 3. Rajvir Dahiya contributed the most research with 34 publications, followed by Sharanjot Saini (29) and Yuichiro Tanaka (29). According to authors’ H-index, Rajvir Dahiya (23) ranked first, followed by Naohiko Seki (22), Sharanjot Saini (20), Yuichiro Tanaka (20), Shahana Majid (20), and Arul M Chinnaiyan (20). Rajvir Dahiya made significant contributions, with his research emphasizing the regulatory roles of miRNAs in the proliferation and metastasis of PC (Noonan et al., 2009; Saini et al., 2011). Co-cited authors are those cited together in one or more publications, used to identify key researchers in a particular academic field. Supplementary Table S2 shows that Rebecca L Siegel (978) had the most co-citations, followed by David P Bartel (451) and Ahmedin Jemal (394). These scholars have established the groundwork for research on ncRNAs in PC.

**TABLE 3:** Top 10 active authors of ncRNA research in PC from 2004 to 2023.

Rank	Author	Counts	H-index	Total citations
1	Rajvir Dahiya	34	23	2,982
2	Sharanjot Saini	29	20	2,267
3	Yuichiro Tanaka	29	20	2,559
4	Shahana Majid	28	20	2,355
5	Varahram Shahryari	24	18	1,931
6	Soichiro Yamamura	24	18	1,976
7	Tapio Visakorpi	24	19	2,898
8	Naohiko Seki	24	22	1,782
9	Arul M Chinnaiyan	22	20	5,096
10	Ming Chen	22	19	929

The data were extracted using CiteSpace.

### 3.4 Analysis of journals

Journal analysis provides researchers with prioritized options for submitting their work and accessing articles within a specific field. A total of 556 academic journals included articles on PC-related ncRNAs. The top 10 journals included 658 papers in the field, accounting for 22.30% of all publications (Table 4). *PLoS One* (102) was the most published journal, followed by *Prostate* (97), *Oncotarget* (95), *Oncogene* (61), and *Scientific Reports* (60). *Cancer Research* (6,656), *PLoS One* (6,567), and *Oncogene* (5,663) were the top three most frequently cited journals.

**TABLE 4:** Top 10 active journals of ncRNA research in PC from 2004 to 2023.

Rank	Journal	Counts	H-index	Total citations	IF (2023)
1	*PLoS One*	102	49	6,567	2.9
2	*Prostate*	97	42	5,343	2.6
3	*Oncotarget*	95	49	5,408	NA
4	*Oncogene*	61	40	5,663	6.9
5	*Scientific Reports*	60	26	1,627	3.8
6	*Oncology Letters*	54	20	941	2.5
7	*Cancers*	48	13	521	4.5
8	*European Review for Medical and Pharmacological Sciences*	48	18	869	NA
9	*Cancer Research*	47	41	6,656	12.5
10	*International Journal of Molecular Sciences*	46	15	680	4.9

The data were extracted using CiteSpace.

The top 20 active co-cited journals are presented in Supplementary Table S3. Among them, *Cancer Research* (5,615) had the most co-citations, followed by *Oncogene* (3,325), *PLoS One* (3,190), *Cell* (3,034), and *Proceedings of the National Academy of Sciences* (2,720). In addition, to investigate the evolutionary relationship between knowledge topics in directly citing journals and cited journals, we used CiteSpace to draw a dual-map overlay of journals, in which the topics of directly citing journals are distributed on the left and the topics of cited journals are represented on the right. The analysis presented in Supplementary Figure S1 revealed a prominent citation pathway, indicating that research published in Molecular/Biology/Genetics journals was predominantly referenced by studies from Molecular/Biology/Immunology journals.

### 3.5 Co-citation analysis of references

**FIGURE 4:**
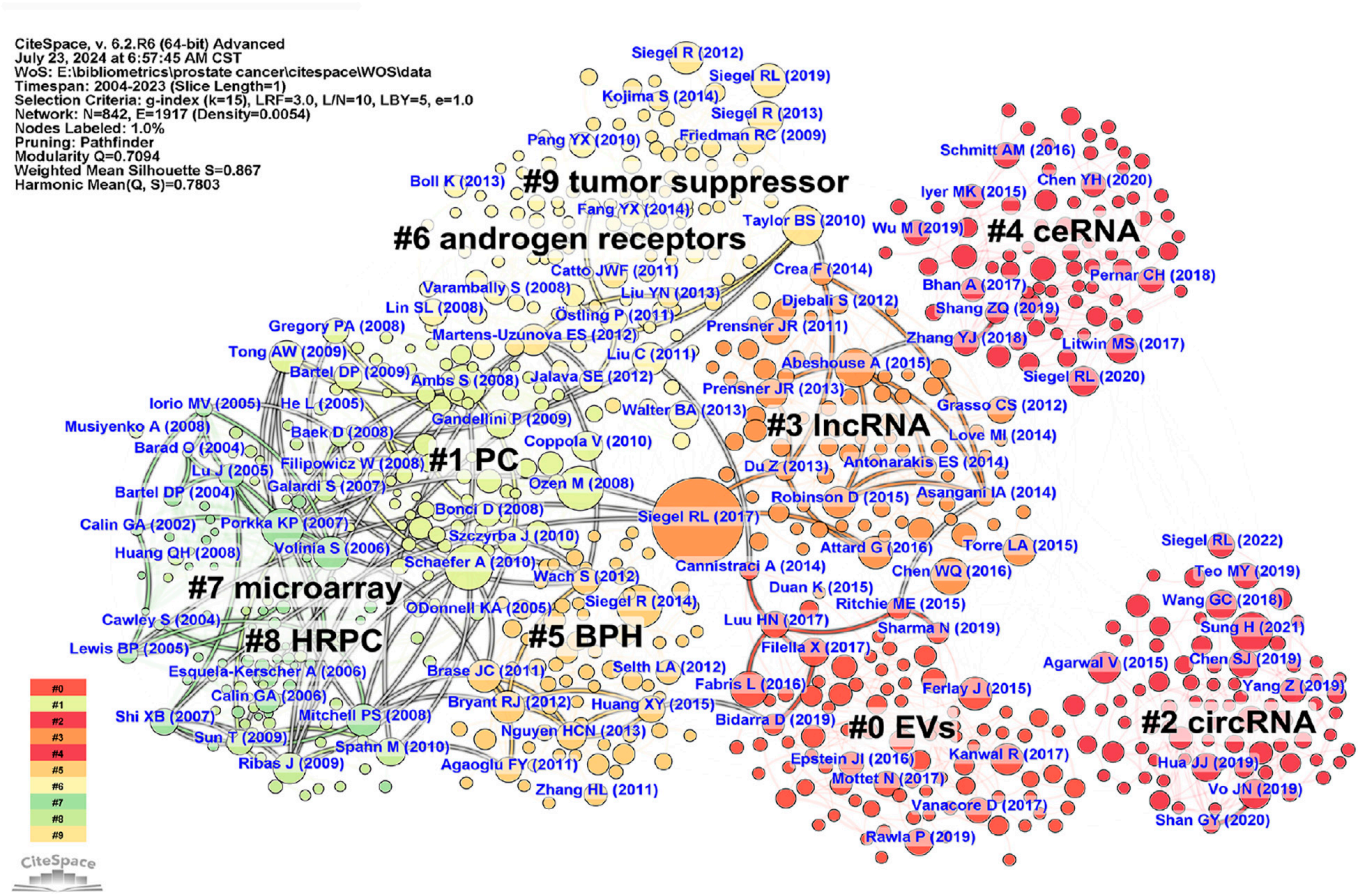
The network map of co-cited references related to ncRNA research in PC from 2004 to 2023. Each node represents a filtered co-cited reference, with the size of the node indicating its co-citation frequency. Each link between two nodes indicates a co-citation relationship between the respective references. Both the data and figures were generated using CiteSpace.

To track the knowledge structure and research trends in the field of PC-related ncRNAs, we conducted a co-citation analysis of the references from 2,951 articles with CiteSpace. Supplementary Table S4 shows the top 10 frequently co-cited references. Among them, “(Siegel et al., 2017)” was the most co-cited reference, with 406 citations. Subsequently, the clustering analysis of CiteSpace was performed to construct a map of co-cited references, which consisted of 842 filtered nodes, 1917 connections, and 10 clusters (Figure 4). The modularity Q score was 0.7094 (>0.3), and the mean silhouette S was 0.867 (>0.7), demonstrating both a significant clustering structure and a highly homogeneous clustering network. Supplementary Figure S2 displays the timeline view of co-cited references, highlighting the evolution of each cluster over time. The largest cluster was “EVs” (cluster #0), followed by “PC” (cluster #1) and “circRNA” (cluster #2). According to the results of cluster analysis, “microarray” (cluster #7), “PC” (cluster #1), and “HRPC” (cluster #8) represented the early knowledge base in the field of PC-related ncRNAs in the past 20 years, while “EVs” (cluster #0), “circRNA” (cluster #2), and “ceRNA” (cluster #4) reflected the current research direction.

Furthermore, through the burst detection function of CiteSpace, we explored the top 15 co-cited references with strong citation bursts (Table 5). The paper that first exhibited strong citation burst over the past 20 years was “*A microRNA expression signature of human solid tumors defines cancer gene targets,*” authored by Stefano Volinia and published in 2006. The paper titled “*microRNA expression in human prostate cancer*” published in 2008 received the strongest citation burst (46.12), followed by “Kati P Porkka, 2007” (42.48), and “Stefan Ambs, 2008” (38.65). These papers constitute significant events in the current knowledge base of PC-related ncRNA research.

**TABLE 5:** Top 15 co-cited references with strong citation bursts related to ncRNA research in PC from 2004 to 2023.

Rank	Co-cited reference	Author	Journal	Year	Burst strength
1	*A microRNA expression signature of human solid tumors defines cancer gene targets*	Stefano Volinia	*Proc Natl Acad Sci U S A*	2006	33.29
2	*MicroRNA expression profiling in prostate cancer*	Kati P Porkka	*Cancer Res*	2007	42.48
3	*Widespread deregulation of microRNA expression in human prostate cancer*	M Ozen	*Oncogene*	2008	46.12
4	*Genomic profiling of microRNA and messenger RNA reveals deregulated microRNA expression in prostate cancer*	Stefan Ambs	*Cancer Res*	2008	38.65
5	*Circulating microRNAs as stable blood-based markers for cancer detection*	Patrick S Mitchell	*Proc Natl Acad Sci U S A*	2008	24.27
6	*MicroRNA profile analysis of human prostate cancers*	A W Tong	*Cancer Gene Ther*	2009	21.29
7	*miR-21: an androgen receptor-regulated microRNA that promotes hormone-dependent and hormone-independent prostate cancer growth*	Judit Ribas	*Cancer Res*	2009	19.19
8	*Diagnostic and prognostic implications of microRNA profiling in prostate carcinoma*	Annika Schaefer	*Int J Cancer*	2010	38.55
9	*Integrative genomic profiling of human prostate cancer*	Barry S Taylor	*Cancer Cell*	2010	31.91
10	*Cancer statistics, 2013*	Rebecca L. Siegel	*CA Cancer J Clin*	2013	24.98
11	*Cancer statistics, 2014*	Rebecca L. Siegel	*CA Cancer J Clin*	2014	32.53
12	*Cancer Statistics, 2017*	Rebecca L. Siegel	*CA Cancer J Clin*	2017	31.19
13	*The Molecular Taxonomy of Primary Prostate Cancer*	Cancer Genome Atlas Research Network	*Cell*	2015	26.97
14	*Cancer statistics in China, 2015*	Wanqing Chen	*CA Cancer J Clin*	2016	21.43
15	*Prostate cancer*	Gerhardt Attard	*Lancet*	2016	20.94

The data were extracted using CiteSpace.

### 3.6 Analysis of keywords

**FIGURE 5:**
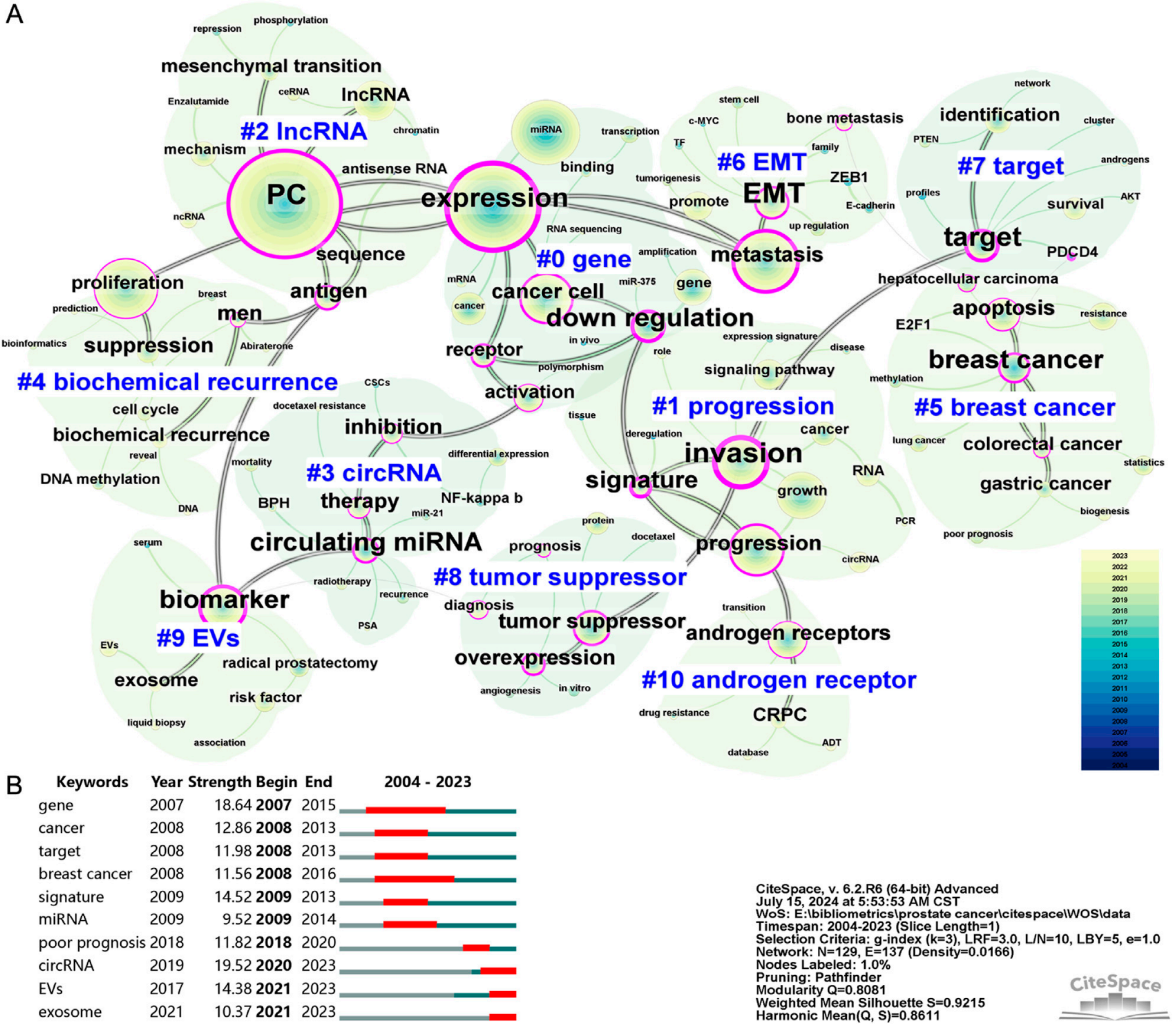
The co-occurrence map **(A)** and burst detection **(B)** of keywords related to ncRNA research in PC from 2004 to 2023. Each node represents a filtered co-occurring keyword, with the size of the node indicating its co-occurrence frequency. Each link between two nodes indicates a co-occurrence relationship between the respective keywords. Nodes with purple rings indicate higher centrality, highlighting their importance within the network. Both the data and figures were generated using CiteSpace.

Identifying the high-frequency keywords in research on PC-associated ncRNAs facilitates the rapid recognition of the hot topics of interest within the academic community. Supplementary Table S5 lists the top 50 most frequent keywords, with the 15 highest-ranking ones being “PC” (1938), “expression” (1,258), “miRNA” (809), “proliferation” (688), “metastasis” (632), “progression” (521), “growth” (492), “cancer cell” (490), “invasion” (447), “gene” (360), “biomarker” (352), “lncRNA” (329), “androgen receptors” (284), “cancer” (268), and “apoptosis” (265). Among these high-frequency keywords, those pertaining to ncRNAs include “miRNA” (809), “lncRNA” (329), and “circRNA” (82). The co-occurrence analysis of keywords helps elucidate the relationships between different knowledge points in PC-associated ncRNA research, thereby contributing to the construction of a knowledge map for the field. To obtain a better network visualization of the keyword co-occurrence, we merged similar keywords from 2,951 articles, resulting in a co-occurrence map with 129 filtered nodes, 137 connections, and 11 clusters (Figure 5A). The modularity Q score was 0.8081, and the mean silhouette S reached 0.9215, indicating the highly convincing keyword network. In addition, we presented a visualization of the evolution of keywords in different clusters over time in the form of a timeline view (Supplementary Figure S3). “gene” (cluster #0) was the largest cluster, encompassing keywords such as “miRNA,” “mRNA,” “polymorphism,” “amplification,” “receptor.” and “activation.” “progression” (cluster #1) was the second largest cluster, including keywords such as “invasion,” “growth,” “signature,” “circRNA,” and “cancer.” Followed by “lncRNA” (cluster #2), including keywords such as “mechanism,” “ceRNA,” “mesenchymal transition,” “chromatin,” and “sequence.”

Burst detection of keywords is employed to identify new or rapidly increasing keywords, which may represent cutting-edge topics or novel areas of research. By using CiteSpace, we obtained the top 10 keywords with the strongest bursts (Figure 5B). The top three keywords with the strongest burst were “circRNA” (19.52), “gene” (18.64), and “signature” (14.52). Notably, the keywords “circRNA,” “EVs,” and “exosome” continued to show significant burst activity in 2023, indicating that these topics are key directions for future research. In addition, we extracted all miRNAs, lncRNAs, and circRNAs from the keywords, and merged and standardized similar miRNAs. Table 6 lists the frequently occurring miRNAs, lncRNAs, and circRNAs, all of which have been confirmed to play significant roles in the development of PC.

**TABLE 6:** Important miRNAs, lncRNAs, and circRNAs in PC from 2004 to 2023.

ncRNA		Expression	Gene Target(s)	Function(s)	Reference
miRNA	miR-21	↑	p57Kip2, IRS1, SREBP-1, BTG2, TGFBR2	Cell proliferation, lipogenesis, EMT, migration	Coppola et al. (2013), Mishra et al. (2014a), Mishra et al. (2014b), and Kanagasabai et al. (2022)
	miR-200	↓	SNAI2, Kaiso, ZEB1	EMT	Liu et al. (2013) and Abisoye-Ogunniyan et al. (2018)
	miR-375	↑	SEC23A, YAP1, PTPN4	Cell proliferation, migration, invasion, apoptosis, chemo-resistance	Szczyrba et al. (2011), Wang et al. (2016), and Gan et al. (2022)
	miR-34	↓	AKT, p53	Cell proliferation, migration, invasion, apoptosis, EMT, joint regulation of stem cell compartment	Majid et al. (2013) and Cheng et al. (2014)
	miR-145	↓	MELK, NCAPG, BUB1, CDK1, MYC, RAS, ERG	Cell proliferation, migration, invasion	Hart et al. (2013), Goto et al. (2017), and Iscaife et al. (2018)
	miR-141	↓	CDC42, CDC42EP3, RAC1, ARPC5, CD44, EZH2, TRAF5, TRAF6	Cell proliferation, migration, invasion, CSC properties, EMT, bone metastasis	Huang et al. (2017) and Liu et al. (2017)
	miR-205	↓	SQLE, ΔNp63α, ZEB1/2, PKCε, RHPN2	Cholesterol biosynthesis, basement membrane maintenance, cell proliferation, migration, invasion, apoptosis, EMT, radiosensitivity	Gandellini et al. (2009), Gandellini et al. (2012), El Bezawy et al. (2019), Jiang et al. (2019), and Kalogirou et al. (2021)
	miR-1	↓	SNAI2, FN1, LASP1, XPO6, TWIST1, E2F5, PFTK1	Cell proliferation, EMT, bone metastasis	Hudson et al. (2012), Liu et al. (2013), Chang et al. (2015), and Li et al. (2018a)
	miR-143	↓	AKT1, KLK2, KRAS	Cell proliferation, migration, EMT, chemo-resistance	Xu et al. (2011), Chu et al. (2016), and Armstrong et al. (2023)
	miR-17	↑	TIMP3	Cell proliferation, migration, invasion	Yang et al. (2013) and Stoen et al. (2021)
	miR-182	↑	MITF	Cell proliferation, migration, invasion, apoptosis, EMT	Wang et al. (2018) and Stafford and McKenna (2023)
lncRNA	lncRNA PCA3	↑			
	lncRNA HOTAIR	↑	EZH2/miR-193a, REST, AR	Cell proliferation, migration, invasion, apoptosis, trimethylation, neuroendocrine differentiation	Zhang et al. (2015), Ling et al. (2017), and Chang et al. (2018)
	lncRNA PCAT1	↑	PHLPP/FKBP51/IKKα, miR-145-5p/FSCN1	Cell proliferation, migration, invasion, apoptosis	Xu et al. (2017) and Shang et al. (2019)
	lncRNA MALAT1	↑	miR-423-5p	Cell proliferation, migration, invasion	Ren et al. (2013) and Ferri et al. (2022)
	lncRNA PVT1	↑	PRC2, miR-15a-5p/KIF23	Cell proliferation, migration, invasion, apoptosis	Videira et al. (2021)
	lncRNA UCA1	↑	miR-143/MYO6, miR-331-3p/EIF4G1, miR-204/CXCR4	Cell proliferation, apoptosis, radiosensitivity	He et al. (2019), Hu and Yang (2020), and Yu et al. (2020)
	lncRNA H19	↑	PRC2	Cell proliferation, invasion, chemo-resistance, histone modification, DNA methylation, CSC properties, neuroendocrine differentiation, chemo-resistance	Singh et al. (2021)
	lncRNA NEAT1	↑	CYCLINL1/CDK19, PSMA, CDC5L/AGRN	Cell proliferation, bone metastasis, therapeutic resistance	Chakravarty et al. (2014), Li et al. (2018b), and Wen et al. (2020)
	lncRNA GAS5	↓	miR-18a, miR-21/PDCD4/PTEN, miR-1284/AKT	Cell proliferation, apoptosis, radiosensitivity	Yang et al. (2019) and Zhu et al. (2019)
	lncRNA MEG3	↓	miR-9-5p/QKI-5, miR-9-5p/NDRG1	Cell proliferation, migration, invasion, apoptosis	Wu et al. (2019) and Lian et al. (2024)
circRNA	Hsa_circ_0003258	↑	miR-653-5p/ARHGAP5, IGF2BP3/HDAC4	migration, invasion, EMT	Yu et al. (2022b)
	circSMARCC1	↑	miR-1322/CCL20/CCR6	Cell proliferation, migration, invasion, EMT, TAMs infiltration	Xie et al. (2022)
	circSPON2	↑	miR-331-3p/PRMT5/CAMK2N1	Cell proliferation, migration, invasion	Yao et al. (2022)
	circARHGAP29	↑	IGF2BP2/c-Myc/LDHA	Chemo-resistance, glycolytic metabolism	Jiang et al. (2022)
	circEXOC6B	↓	AKAP12	Migration, invasion	Zhang et al. (2023)
	circCEMIP	↑	miR-1248/TM9SF4	Migration, invasion, autophagy, anoikis resistance	Zhang et al. (2018) and Yu et al. (2022a)
	circPDE5A	↓	WTAP/EIF3C/MAPK	Migration, invasion, methylation	Ding et al. (2022)

The data were extracted using CiteSpace.

## 4 Discussion

### 4.1 General information

With the rapid development of information technology and increased medical academic papers, bibliometrics has been applied in various medical fields and plays an irreplaceable role in quantitative analysis. In this study, we retrieved publications indexed in WoSCC over the past 20 years and excluded records unrelated to ncRNA research in PC. Ultimately, 2,951 published articles were included in our bibliometric analysis. Between 2009 and 2023, there was a marked increase in both the total number of publications and citations concerning ncRNA research in PC, underscoring its emergence as a rapidly expanding area of interest. China had the highest number of publications and the second-highest number of citations, which shows its significant contribution in this field. As the most influential country, the United States collaborated with 26 countries/regions and maintained its closest partnership with China. In terms of institutional distribution, Fudan University, which had the highest number of publications, primarily collaborated with the Naval Medical University, Tongji University, and Chinese Academy of Sciences. The research focus of these institutions mainly revolves around understanding the mechanisms by which miRNAs (Li et al., 2014; Xiang et al., 2015), lncRNAs (Yao et al., 2020), and circRNAs (Kong et al., 2021) contribute to the progression of PC. According to the author analysis, Rajvir Dahiya was the most active and influential author in this field.

Additionally, we identified the top 10 most cited articles in ncRNA research related to PC. The most cited paper was published in *Nature* by Gao et al. (2009), which was groundbreaking in linking changes in miR-23a/b expression and glutamine metabolism to PC progression and proposed that c-Myc plays a crucial role in regulating crucial metabolic pathways in PC. The paper with the highest annual citation frequency was published by Vo et al. (2019) in 2019 in *Cell*, which highlighted the potential of circRNAs as diagnostic biomarkers and therapeutic targets for PC.

### 4.2 Knowledge base

We further explored the theoretical foundations of PC-related ncRNA research through co-citation analysis of the references. Through CiteSpace clustering analysis, all co-cited references were categorized into a network map containing 10 clusters with diverse labels, where “EVs” (cluster #0), “circRNA” (cluster #2), and “ceRNA” (cluster #4) reflected the current research trends. The top 10 frequently co-cited references included four epidemiological studies and six foundational studies. These epidemiological findings consistently highlighted PC as a leading cause of cancer-related deaths in men and a significant public health concern in China (Siegel et al., 2014; Chen et al., 2016; Siegel et al., 2017; Sung et al., 2021). Among the six foundational studies, four investigated microRNA profiles in PC (Porkka et al., 2007; Ambs et al., 2008; Ozen et al., 2008; Schaefer et al., 2010), whereas the other two focused on exploring the molecular and genomic characteristics of the disease (Taylor et al., 2010; Network, 2015). Moreover, we identified the top 15 popular references in PC-related ncRNA research based on citation bursts, which include most of the highly co-cited references mentioned above. It is worth noting that the study by Mitchell et al. (2008) underscored the potential of circulating miRNAs in serum or plasma as stable blood-based biomarkers for cancer classification and prognostication, specifically demonstrating that miR-141 effectively distinguished PC patients from healthy controls. This research provided the first strong evidence supporting the diagnostic utility of circulating miRNAs in PC. Ribas et al. (2009) were the first to discover that high expression of miR-21 promoted the growth of PC and conferred castration resistance, while inhibition of miR-21 reduced androgen-driven proliferation of PC cells. This study provided crucial molecular insights into targeting miRNAs for the treatment of PC. In summary, co-citation analysis of the references enables us to better understand the knowledge structure of PC-related ncRNAs and to identify the core references within this field.

### 4.3 Research hotspots

To explore the research hotspots in PC-related ncRNAs, we analyzed the keywords from 2,951 articles. High-frequency keywords with co-occurrence rates exceeding 500 included “PC,” “expression,” “miRNA,” “proliferation,” “metastasis,” and “progression,” indicating that research primarily focused on the molecular mechanisms of miRNAs in PC proliferation and metastasis. Commonly mentioned miRNAs included miR-21, miR-200, miR-375, miR-34, miR-145, miR-141, miR-205, miR-1, miR-143, miR-17, and miR-182. Aside from “miRNA,” “lncRNA” and “circRNA” were also high-frequency keywords associated with ncRNAs, highlighting their significant relevance in the field of PC. We also used CiteSpace for keyword burst detection, which revealed that recent emergent keywords include “circRNA,” “EVs,” and “exosome,” all of which represent cutting-edge areas in ncRNA research related to PC. Here, we provide a brief discussion on the potential of circRNAs and EVs in PC.

#### 4.3.1 circRNAs

Early detection of PC remains crucial in reducing cancer-related mortality in men. The identification of potential cancer biomarkers significantly enhances the diagnosis and ongoing monitoring of PC, thereby improving patient outcomes. PSA liquid biopsy is the most common method for PC detection. However, its limited specificity in distinguishing between benign prostatic hyperplasia (BPH) and PC poses significant challenges for early detection and diagnosis (Stenman et al., 1999). circRNA was first discovered in RNA viruses in 1976 and was initially considered as splicing noise based on its biogenesis (Sanger et al., 1976). Until recent years, the rapid development of RNA sequencing technology has led to the identification of numerous circRNAs in eukaryotes, offering promising insights for the discovery of novel PC-related biomarkers based on liquid biopsies (Wang et al., 2014; Maass et al., 2017). As a newly discovered, abundant, and conserved class of ncRNAs, circRNAs originate from precursor mRNA and are characterized by their covalently closed loop structure formed through backsplicing (Jeck and Sharpless, 2014). With a unique structure lacking free ends, circRNAs are less exposed to exoribonuclease degradation (Jeck and Sharpless, 2014). This stability enables their reliable presence in plasma, urine, and saliva, reinforcing their potential as ideal circulating materials for liquid biopsies. Several investigations have emphasized the potential of circRNAs as biomarkers for PC. By utilizing exome capture RNA sequencing, Vo et al. (2019) established the MiOncoCirc database composed of circRNAs detected in tumor tissues, and identified 1,092 circRNAs in urine samples from PC patients. Chen et al. (2019) performed ultra-deep non-poly-A RNA sequencing on tumor samples from 144 patients with localized PC, revealing 76,311 distinct circRNAs. Among these, 171 circRNAs were identified as being essential for the proliferation of PC cells (Chen et al., 2019). These transcriptional profiles contribute to advancing the application of circRNAs in the diagnostic medicine of PC.

Furthermore, since circRNAs were first characterized as transcriptional regulators that control the miRNA-mRNA axis, an increasing body of research have preliminarily confirmed their critical roles in the epigenetic regulation associated with PC (Hansen et al., 2013; Zhou et al., 2021). Yu et al. (2022b) first observed the overexpression of hsa_circ_0003258 in human PC tissues, which was associated with the aggressive progression of the disease. Subsequent investigation revealed that hsa_circ_0003258 enhances the expression of Rho GTPase activating protein 5 by sponging miR-653-5p and forming a complex with insulin-like growth factor 2 mRNA binding protein 3 to stabilize HDAC4 mRNA. This interaction activates the ERK signaling pathway, accelerating the epithelial-mesenchymal transition (EMT) and ultimately promoting PC metastasis (Yu et al., 2022b). Xie et al. (2022) found that circSMARCC1 was significantly upregulated in the plasma and tissues of PC patients, promoting tumor proliferation and metastasis. Mechanistically, circSMARCC1 sponges miR-1322 to regulate the expression of CC-chemokine ligand 20 (CCL20), which activates PC cell proliferation and EMT. Additionally, circSMARCC1 induces the infiltration of tumor-associated macrophages and M2 polarization through the CCL20-CCR6 axis, thereby facilitating the progression of PC. Yu et al. (2022a) reported that the upregulation of circCEMIP in PC tissues promoted the invasion and metastasis of PC cells. CircCEMIP functions as a ceRNA for miR-1248, reducing the inhibitory effects of miR-1248 on its downstream target, transmembrane 9 superfamily member 4, which induces mTOR phosphorylation-mediated anoikis resistance (Yu et al., 2022a). In addition to serving as miRNA sponges, circRNAs interact with RNA-binding proteins and even participate in protein-coding processes, thus modulating the pathological processes of PC. Feng et al. (2019) identified that circ0005276, upregulated in PC tissues, promoted the proliferation, migration, and EMT of PC cells by interacting with the RNA-binding protein FUS to activate the transcription of X-linked inhibitor of apoptosis protein. Wang et al. (2024) discovered that the protein-coding circRNA circCCDC7 was significantly downregulated in PC patients and indicated that it upregulates FLRT3 by encoding the secretory protein circCCDC7-180aa, thereby inhibiting PC cell activity and suggesting its potential role as a tumor suppressor in PC. These studies highlight the critical involvement of circRNAs in the proliferation, metastasis, and treatment resistance associated with PC. Further exploration of circRNAs will enhance our understanding of the pathogenic mechanisms underlying PC and provide therapeutic strategies for future clinical applications.

#### 4.3.2 EVs

EVs are small vesicles naturally released by all cell types into the extracellular space, initially believed to be membranous structures derived from cells for the purpose of clearing metabolic waste (van Niel et al., 2018). Subsequently, extensive research has uncovered that EVs, particularly exosomes, participate in various intercellular communication processes and have emerged as a prominent area of interest in biomedicine and bioengineering (van Niel et al., 2018; Kalluri and LeBleu, 2020). As nanoparticles encapsulated by a lipid bilayer membrane, EVs function as optimal carriers for safely transporting ncRNAs, playing a pivotal role in PC progression and metastasis by transferring these biomolecules to target cells (Mugoni et al., 2022). Liquid biopsies based on ncRNAs in EVs provide a non-invasive alternative to tissue biopsies, offering an abundant source of biological material and enabling the identification of potential tumor markers that can inform the staging and risk prognosis of PC (Hamed et al., 2024; Hu et al., 2024). The miRNA family represents one of the most frequently identified classes of ncRNAs linked to EVs. In a study comparing blood samples from 102 PC patients with those of 50 healthy controls, Souza et al. (2017) found that miR-200b levels in circulating EVs were associated with PSA levels exceeding 10 ng/mL and bone metastasis, whereas miR-200c expression was associated with Gleason score. Similarly, miR-424 (Albino et al., 2021) and miR-1246 (Bhagirath et al., 2018) in circulating EVs from PC patients were correlated with the metastatic spread of tumor cells. Some researchers have attached importance to the value of EVs in predicting the efficacy of drug treatment and radiotherapy in PC patients. For example, Guo et al. (2020) identified miR-423-3p as a potential biomarker for early prediction of castration resistance by analyzing plasma exosomal miRNAs in PC patients and those with CRPC after ADT. Wang et al. (2016) showed that elevated levels of miR-375 were involved in the chemo-resistance to docetaxel in metastatic CRPC patients and were significantly associated with their overall survival. Additionally, Yu et al. (2018) found that 57 miRNAs in serum exosomes exhibited significant changes following carbon ion radiotherapy in localized PC patients, with miR-654-3p and miR-379-5p emerging as potential predictors of therapeutic response to this treatment.

It is evident that PC-derived EVs function as crucial carriers of ncRNAs, facilitating the progression of PC through intercellular communication, including tumor proliferation, metastasis, angiogenesis, immune evasion, and drug resistance (Hu et al., 2024). Recent studies have identified several miRNAs, lncRNAs, and circRNAs within PC-derived EVs as potential inhibitory targets in PC therapy, offering new insights into precision medicine for this disease (Aghdam et al., 2019; Xu et al., 2019; Zhou et al., 2021). Additionally, compared to traditional delivery vehicles, EVs demonstrate superior biocompatibility and delivery efficiency, and their intrinsic advantages of naturally homing to tumor cells offer prospects for PC therapeutic strategies targeting EVs (Hu et al., 2024). Inhibiting the secretion of PC-derived EVs could be a critical step in reducing communication within the tumor microenvironment. Urabe et al. (2020) conducted a high-throughput miRNA-based screening using the ExoScreen assay and identified miR-26a as a regulator of EV secretion in PC cells. The associated mechanism involved the modulation of the SHC4, PFDN4, and CHORDC1 genes. Moreover, limited data confirmed that certain normal cells exhibit antitumor activity against PC. For instance, adipose-derived stromal cells release EVs containing miR-145, which suppress the proliferation of PC cells and induce apoptosis (Takahara et al., 2016). Exosomal miR-205 and miR-99b-5p derived from human bone marrow mesenchymal stem cells inhibit the proliferation, invasion, and migration of PC cells (Jiang et al., 2019). A deeper understanding of the roles of ncRNAs in EVs secreted by different cell types in PC paves the way for novel therapeutic strategies based on engineered EVs.

### 4.4 Limitations

In this study, we conducted the first bibliometric analysis using data from the WoSCC to objectively assess the research trends and current landscape in the field of PC-related ncRNAs. While our approach offers valuable insights, it is important to recognize certain limitations. First, although WoSCC is widely considered the premier database for bibliometric studies, there remains the possibility that some relevant articles were not captured, potentially introducing minor biases in our findings. Moreover, our analysis was based on data from publications spanning 2004 to 2023, limiting its ability to reflect the most recent advances in the field. Third, the nature of bibliometric analysis tends to prioritize highly cited mainstream research, potentially overlooking less cited but innovative studies. Nevertheless, we believe these limitations do not substantially diminish the relevance or contributions of the publications included in our analysis to the field.

## 5 Conclusion

This study presented a comprehensive bibliometric analysis of 2,951 articles on PC-related ncRNAs over the past 20 years. The global publication output in this field had seen rapid growth since 2011, indicating its emergence as a focal point of scholarly attention. China led in publication output, while the United States was the most influential nation, engaging in collaborating with 26 other countries. Rajvir Dahiya stood out as the most prolific and influential author. The research landscape of PC- related ncRNAs was predominantly focused on elucidating the molecular mechanisms by which miRNAs drive PC proliferation and metastasis. Additionally, circRNAs and EVs are emerging as pivotal areas of future exploration, offering promising avenues for advancing the precise diagnosis and targeted treatment of PC.

## Data Availability

The original contributions presented in the study are included in the article/Supplementary Material, further inquiries can be directed to the corresponding author.
