# Supplementary material for: Global research landscape and emerging trends of non-coding RNAs in prostate cancer: a bibliometric analysis
Source: Front Pharmacol. 2025 Jan 7;15:1483186. doi: 10.3389/fphar.2024.1483186 (PMC11753231; doi:10.3389/fphar.2024.1483186)
Supplement: Supplementary file 1 [file Table1.docx]

Supplementary Table S1 Top 10 highly-cited articles of ncRNA research in PC from 2004 to 2023.

| Rank | Title | Total citation | Average citation | Author | Journal | Year |
| --- | --- | --- | --- | --- | --- | --- |
| 1 | *c-Myc suppression of miR-23a/b enhances mitochondrial glutaminase expression and glutamine metabolism* | 1641 | 109.40 | Ping Gao | *Nature* | 2009 |
| 2 | *The microRNA miR-34a inhibits prostate cancer stem cells and metastasis by directly repressing CD44* | 1153 | 88.69 | Can Liu | *Nat Med* | 2011 |
| 3 | *Molecular Interplay of the Noncoding RNA ANRIL and Methylated Histone H3 Lysine 27 by Polycomb CBX7 in Transcriptional Silencing of INK4a* | 1100 | 78.57 | Kyoko L Yap | *Mol Cell* | 2010 |
| 4 | *The Landscape of Circular RNA in Cancer* | 1077 | 215.40 | Josh N Vo | *Cell* | 2019 |
| 5 | *Genomic Loss of microRNA-101 Leads to Overexpression of Histone Methyltransferase EZH2 in Cancer* | 840 | 52.50 | Sooryanarayana Varambally | *Science* | 2008 |
| 6 | *Transcriptome sequencing across a prostate cancer cohort identifies PCAT-1, an unannotated lincRNA implicated in disease progression* | 827 | 63.62 | John R Prensner | *Nat Biotechnol* | 2011 |
| 7 | *The miR-15a-miR-16-1 cluster controls prostate cancer by targeting multiple oncogenic activities* | 810 | 50.63 | Désirée Bonci | *Nat Med* | 2008 |
| 8 | *A novel class of small RNAs: tRNA-derived RNA fragments (tRFs)* | 803 | 53.53 | Yong Sun Lee | *Genes Dev* | 2009 |
| 9 | *MicroRNA expression profiling in prostate cancer* | 743 | 43.71 | Kati P Porkka | *Cancer Res* | 2007 |
| 10 | *Optimized high-throughput microRNA expression profiling provides novel biomarker assessment of clinical prostate and breast cancer biopsies* | 589 | 32.72 | Michael D Mattie | *Mol Cancer* | 2006 |
