# Supplementary material for: Global research landscape and emerging trends of non-coding RNAs in prostate cancer: a bibliometric analysis
Source: Front Pharmacol. 2025 Jan 7;15:1483186. doi: 10.3389/fphar.2024.1483186 (PMC11753231; doi:10.3389/fphar.2024.1483186)
Supplement: Supplementary file 2 [file Table2.docx]

Supplementary Table S2 Top 15 active co-cited authors of ncRNA research in PC from 2004 to 2023.

| Rank | Co-cited author | Co-citations |
| --- | --- | --- |
| 1 | Rebecca L Siegel | 978 |
| 2 | David P Bartel | 451 |
| 3 | Ahmedin Jemal | 394 |
| 4 | Kati P Porkka | 282 |
| 5 | George A Calin | 260 |
| 6 | K J Livak | 250 |
| 7 | Barry S Taylor | 241 |
| 8 | John R Prensner | 239 |
| 9 | Stefano Volinia | 225 |
| 10 | Jun Lu | 211 |
| 11 | Stefan Ambs | 209 |
| 12 | M Ozen | 206 |
| 13 | Annika Schaefer | 206 |
| 14 | Patrick S Mitchell | 183 |
| 15 | Jacques Ferlay | 179 |
