# Supplementary material for: Global research landscape and emerging trends of non-coding RNAs in prostate cancer: a bibliometric analysis
Source: Front Pharmacol. 2025 Jan 7;15:1483186. doi: 10.3389/fphar.2024.1483186 (PMC11753231; doi:10.3389/fphar.2024.1483186)
Supplement: Supplementary file 3 [file Table5.docx]

Supplementary Table S5 Top 50 frequency keywords of ncRNA research in PC from 2004 to 2023.

| Rank | keyword | Frequency | Rank | keyword | Frequency |
| --- | --- | --- | --- | --- | --- |
| 1 | PC | 1938 | 26 | mechanism | 167 |
| 2 | expression | 1258 | 27 | survival | 151 |
| 3 | miRNA | 809 | 28 | protein | 145 |
| 4 | proliferation | 688 | 29 | cancer | 144 |
| 5 | metastasis | 632 | 30 | RNA | 138 |
| 6 | progression | 521 | 31 | suppression | 126 |
| 7 | growth | 492 | 32 | inhibition | 117 |
| 8 | cancer cell | 490 | 33 | antigen | 117 |
| 9 | invasion | 447 | 34 | therapy | 114 |
| 10 | gene | 360 | 35 | receptor | 111 |
| 11 | biomarker | 352 | 36 | risk factor | 106 |
| 12 | lncRNA | 329 | 37 | circulating miRNA | 105 |
| 13 | androgen receptors | 284 | 38 | lung cancer | 102 |
| 14 | cancer | 268 | 39 | transcription | 98 |
| 15 | apoptosis | 265 | 40 | mesenchymal transition | 98 |
| 16 | tumor suppressor | 220 | 41 | differential expression | 98 |
| 17 | signaling pathway | 219 | 42 | radical prostatectomy | 96 |
| 18 | target | 211 | 43 | ncRNA | 95 |
| 19 | EMT | 211 | 44 | overexpression | 93 |
| 20 | identification | 209 | 45 | statistics | 91 |
| 21 | promote | 194 | 46 | signature | 90 |
| 22 | activation | 180 | 47 | diagnosis | 87 |
| 23 | resistance | 180 | 48 | circRNA | 82 |
| 24 | down regulation | 171 | 49 | hepatocellular carcinoma | 74 |
| 25 | breast cancer | 170 | 50 | biochemical recurrence | 71 |
