# Supplementary material for: Global research landscape and emerging trends of non-coding RNAs in prostate cancer: a bibliometric analysis
Source: Front Pharmacol. 2025 Jan 7;15:1483186. doi: 10.3389/fphar.2024.1483186 (PMC11753231; doi:10.3389/fphar.2024.1483186)
Supplement: Supplementary file 4 [file Table3.docx]

Supplementary Table S3 Top 20 active co-cited journals of ncRNA research in PC from 2004 to 2023.

| Rank | Co-cited Journal | Total co-citations | IF（2023） |
| --- | --- | --- | --- |
| 1 | *Cancer Research* | 5615 | 12.5 |
| 2 | *Oncogene* | 3325 | 6.9 |
| 3 | *PLoS One* | 3190 | 2.9 |
| 4 | *Cell* | 3034 | 45.5 |
| 5 | *Proceedings of the National Academy of Sciences* | 2720 | 9.4 |
| 6 | *Nature* | 2633 | 50.5 |
| 7 | *Oncotarget* | 2543 | NA |
| 8 | *Nucleic Acids Research* | 2398 | 16.6 |
| 9 | *Prostate* | 2311 | 2.6 |
| 10 | *European Urology* | 1778 | 25.3 |
| 11 | *CA: A Cancer Journal for Clinicians* | 1672 | 503.1 |
| 12 | *Clinical Cancer Research* | 1659 | 10 |
| 13 | *International Journal of Cancer* | 1653 | 5.7 |
| 14 | *Journal of Biological Chemistry* | 1614 | 4 |
| 15 | *Molecular Cancer* | 1396 | 27.7 |
| 16 | *Cancer Cell* | 1290 | 48.8 |
| 17 | *British Journal of Cancer* | 1268 | 6.4 |
| 18 | *Science* | 1186 | 47.7 |
| 19 | *Nature Genetics* | 1154 | 31.7 |
| 20 | *Nature Reviews Cancer* | 1151 | 72.5 |
