# Supplementary figures and images for: Global research landscape and emerging trends of non-coding RNAs in prostate cancer: a bibliometric analysis
Source: Front Pharmacol. 2025 Jan 7;15:1483186. doi: 10.3389/fphar.2024.1483186 (PMC11753231; doi:10.3389/fphar.2024.1483186)

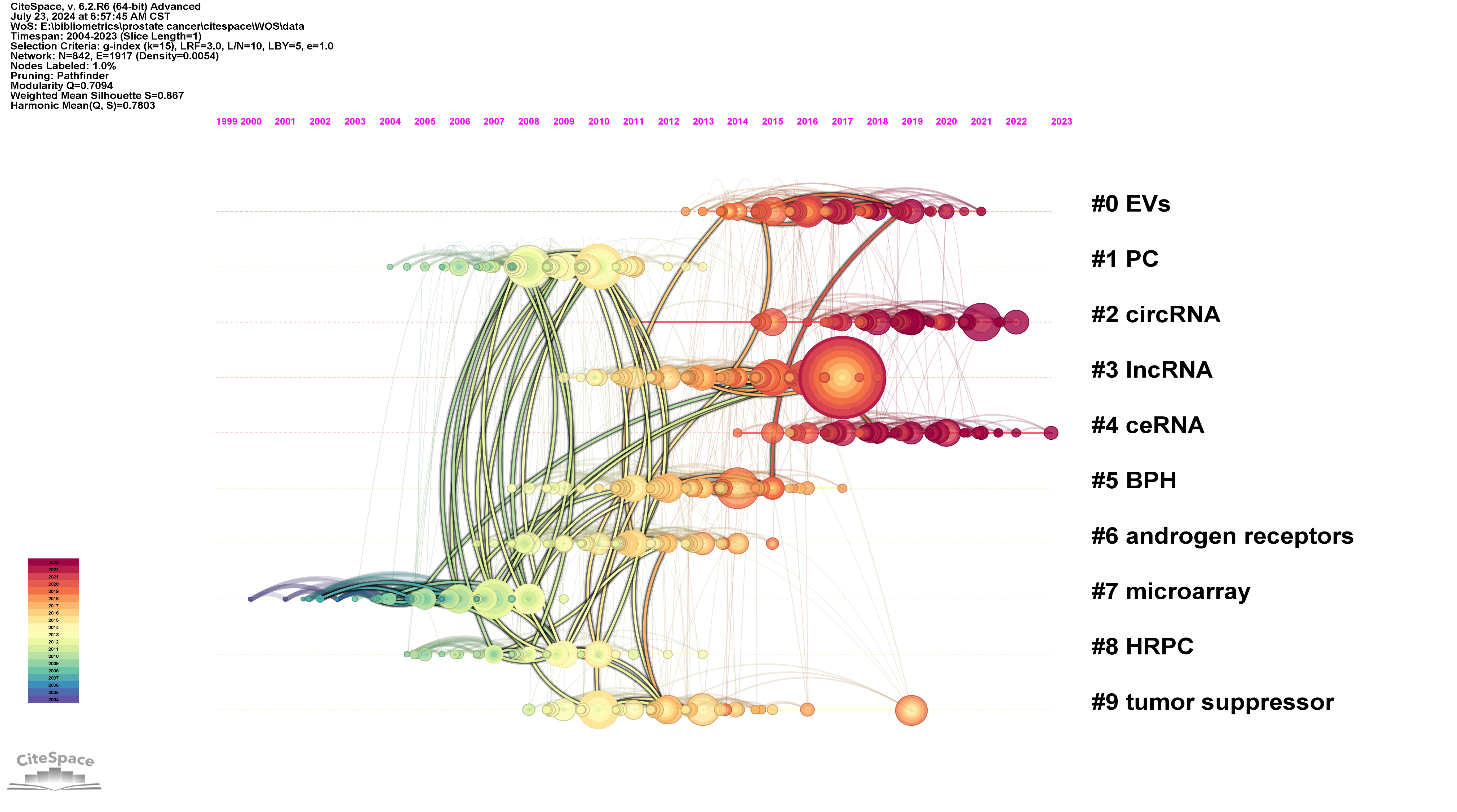

Supplement: Supplementary file 5 [file Image2.png]

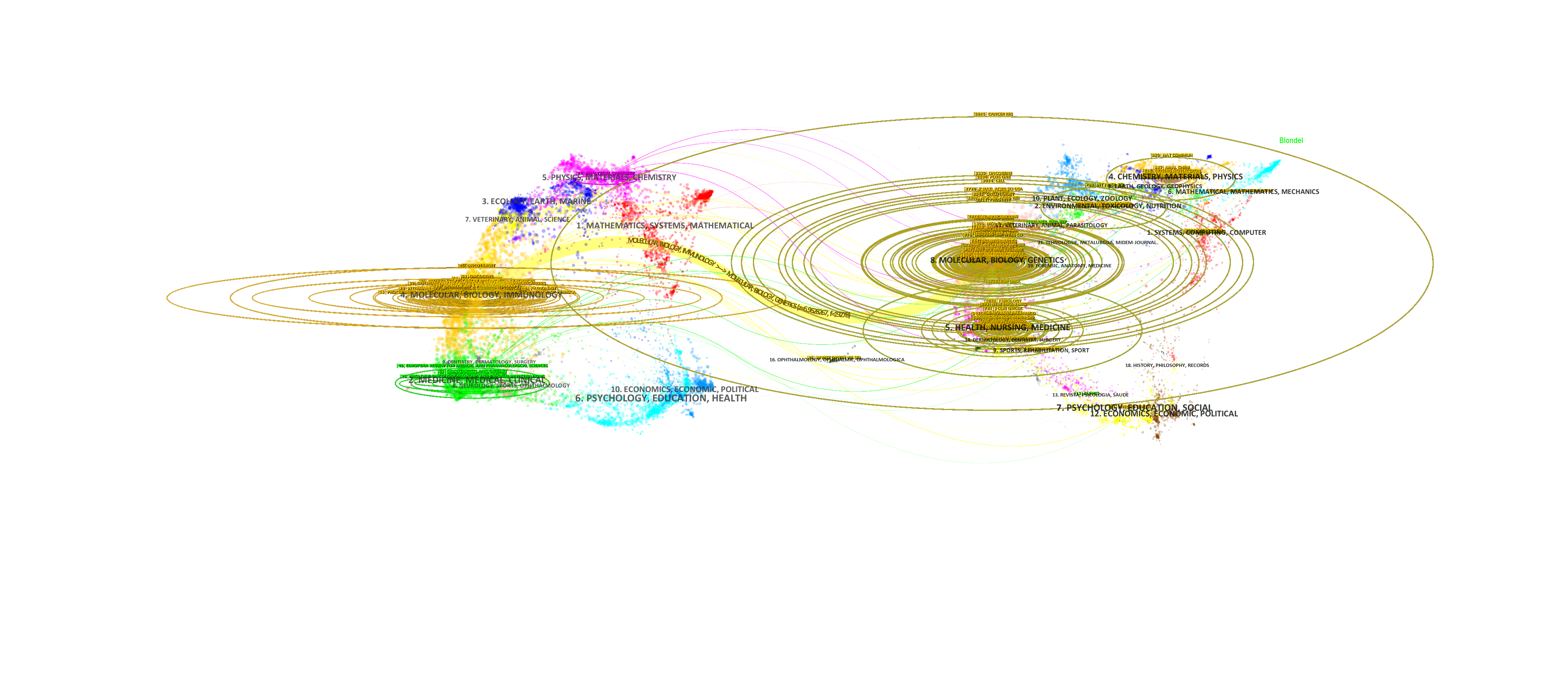

Supplement: Supplementary file 7 [file Image1.png]

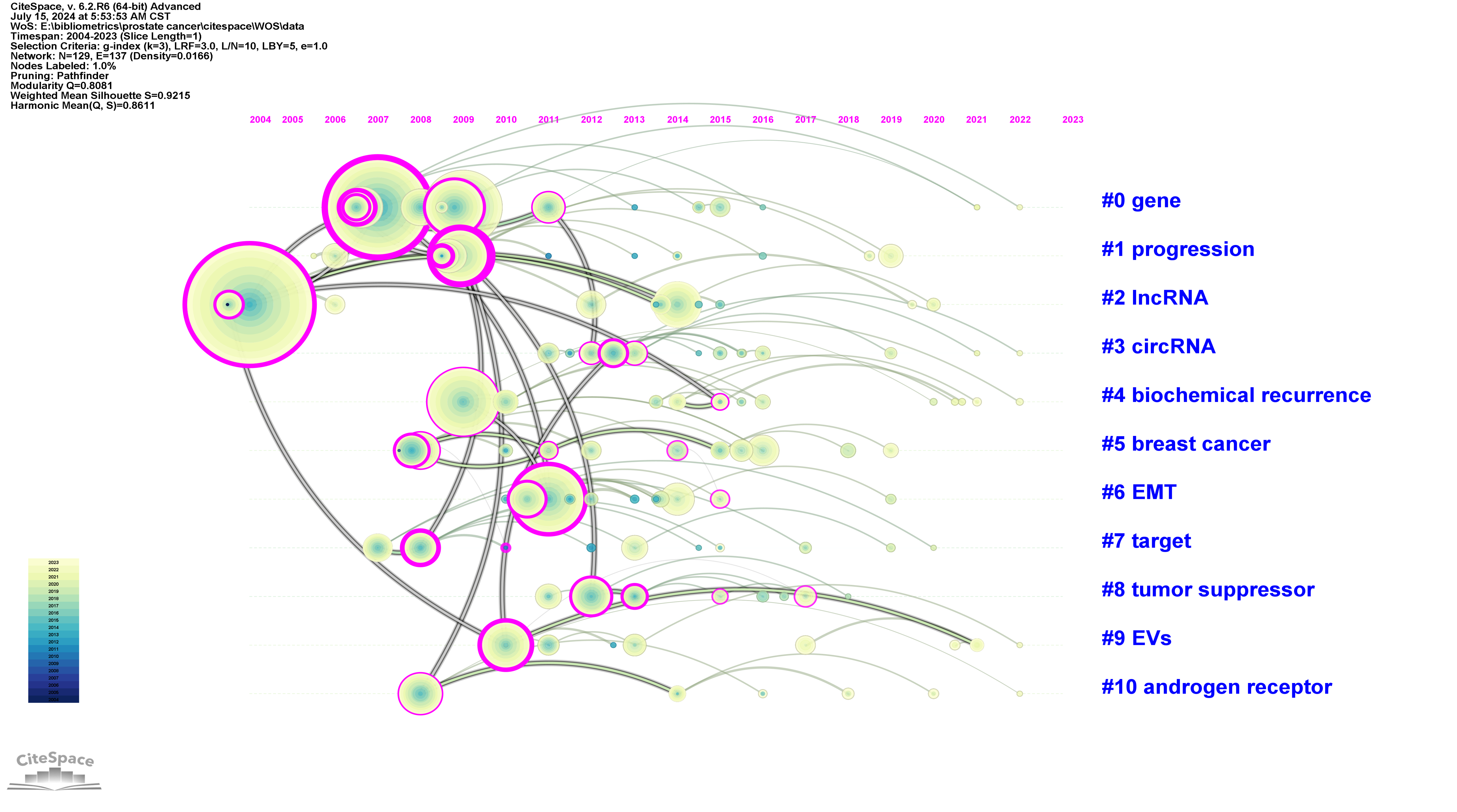

Supplement: Supplementary file 8 [file Image3.png]
