# Supplementary material for: Global research landscape and emerging trends of non-coding RNAs in prostate cancer: a bibliometric analysis
Source: Front Pharmacol. 2025 Jan 7;15:1483186. doi: 10.3389/fphar.2024.1483186 (PMC11753231; doi:10.3389/fphar.2024.1483186)
Supplement: Supplementary file 6 [file Table4.docx]

Supplementary Table S4 Top 10 co-cited references related to ncRNA research in PC from 2004 to 2023.

| Rank | Reference | Author | Journal | Year | Co-citations |
| --- | --- | --- | --- | --- | --- |
| 1 | *Cancer Statistics, 2017* | Rebecca L Siegel | *CA Cancer J Clin* | 2017 | 406 |
| 2 | *Diagnostic and prognostic implications of microRNA profiling in prostate carcinoma* | Annika Schaefer | *Int J Cancer* | 2010 | 110 |
| 3 | *Widespread deregulation of microRNA expression in human prostate cancer* | M Ozen | *Oncogene* | 2008 | 101 |
| 4 | *Cancer statistics, 2014* | Rebecca L Siegel | *CA Cancer J Clin* | 2014 | 100 |
| 5 | *Genomic profiling of microRNA and messenger RNA reveals deregulated microRNA expression in prostate cancer* | Stefan Ambs | *Cancer Res* | 2008 | 85 |
| 6 | *MicroRNA expression profiling in prostate cancer* | Kati P Porkka | *Cancer Res* | 2007 | 80 |
| 7 | *Integrative genomic profiling of human prostate cancer* | Barry S Taylor | *Cancer Cell* | 2010 | 80 |
| 8 | *Global Cancer Statistics 2020: GLOBOCAN Estimates of Incidence and Mortality Worldwide for 36 Cancers in 185 Countries* | Hyuna Sung | *CA Cancer J Clin* | 2021 | 78 |
| 9 | *The Molecular Taxonomy of Primary Prostate Cancer* | The Cancer Genome Atlas Research Network | *Cell* | 2015 | 76 |
| 10 | *Cancer statistics in China, 2015* | Wanqing Chen | *CA Cancer J Clin* | 2016 | 73 |
